# The lysosomal protein cathepsin L is a progranulin protease

**DOI:** 10.1186/s13024-017-0196-6

**Published:** 2017-07-25

**Authors:** Chris W. Lee, Jeannette N. Stankowski, Jeannie Chew, Casey N. Cook, Ying-Wai Lam, Sandra Almeida, Yari Carlomagno, Kwok-Fai Lau, Mercedes Prudencio, Fen-Biao Gao, Matthew Bogyo, Dennis W. Dickson, Leonard Petrucelli

**Affiliations:** 1 Department of Neuroscience, Mayo Clinic, Jacksonville, Florida, 32224 USA; 2 Present Address: Atlantic Health System, Morristown, NJ USA; 3 Present Address: Biomedical Research Institute of New Jersey, Cedar Knolls, NJ USA; 4 Vermont Genetics Network Proteomics Facility, University of Vermont, Burlington, VT USA; 5 Department of Biology, University of Vermont, Burlington, VT USA; 6 Department of Neurology, University of Massachusetts Medical School, Worcester, MA USA; 7 School of Life Sciences, Faculty of Science, The Chinese University of Hong Kong, Shatin, Hong Kong SAR; 8 Department of Chemical and Systems Biology, Stanford University School of Medicine, Stanford, CA USA

**Keywords:** Progranulin, Lysosome, Cathepsin L, Neutrophil elastase, Frontotemporal lobar degeneration, Neuronal ceroid lipofuscinosis

## Abstract

Haploinsufficiency of GRN, the gene encoding progranulin (PGRN), causes frontotemporal lobar degeneration (FTLD), the second most common cause of early-onset dementia. Receptor-mediated lysosomal targeting has been shown to regulate brain PGRN levels, and complete deficiency of PGRN is a direct cause of neuronal ceroid lipofuscinosis (NCL), a lysosomal storage disease. Here we show that the lysosomal cysteine protease cathepsin L (Cat L) can mediate the proteolytic cleavage of intracellular PGRN into poly-granulin and granulin fragments. Further, PGRN and Cat L co-localize in lysosomes of HEK293 cells, iPSC-derived neurons and human cortical neurons from human postmortem tissue. These data identify Cat L as a key intracellular lysosomal PGRN protease, and provides an intriguing new link between lysosomal dysfunction and FTLD.

**Electronic supplementary material:**

The online version of this article (doi:10.1186/s13024-017-0196-6) contains supplementary material, which is available to authorized users.

## Background

Frontotemporal dementia (FTD) is a neurodegenerative disorder resulting from frontotemporal lobar degeneration (FTLD), and is characterized by progressive changes in personality, behavior, and/or language. FTD is the most common cause of early-onset dementia next to Alzheimer’s disease in patients under the age of 65 [1]. Haploinsufficiency of *GRN*, the gene encoding progranulin (PGRN), is responsible for approximately 5–11% and 5% of clinical cases of familial and sporadic frontotemporal dementia (i.e. FTLD-GRN), respectively [2–5]. While complete loss of PGRN is linked to an adult-onset form of neuronal ceroid lipofuscinosis (NCL), a progressive and fatal lysosomal storage disorder [6, 7].

PGRN is a glycoprotein with multiple downstream effects. [8–10]. PGRN levels are regulated by sortilin (SORT1) -mediated endocytosis and lysosomal targeting [11, 12], and PGRN lysosomal trafficking is also regulated by the prosaposin/mannose-6-phosphate receptor (M6PR) pathway independent of SORT1 [13, 14]. In the brain, PGRN is mainly expressed in neurons and activated microglia, and has been reported to have neurotrophic and neuroprotective functions, maintaining neuronal survival and proper neurite outgrowth and branching [9, 15–17]. PGRN contains seven and a half tandem granulin motifs separated by linker regions and proteolytic processing of PGRN results in the formation of several distinct granulin proteins [8–10]. Predominantly extracellular proteases, such as neutrophil elastase [18] and MMP12 [19], have been shown to cleave the secreted form of PGRN into mature, individual granulins A-G, as well as intermediate poly-granulins. However, lysosomal PGRN proteases have yet to be identified. As specific biological roles for full-length PGRN and/or the cleaved granulins remain unclear, it is important to further our understanding of the mechanisms involved in PGRN proteolytic processing.

Cathepsins are cysteine proteases acting within the lysosomal/autophagy pathway. Interestingly, mutations in cathepsins have been linked to NCL pathogenesis in both humans and animal models [20, 21], while cathepsin D (Cat D) levels are elevated in *Grn* ^*−/−*^ mice and FTLD-GRN patients [22, 23]. Additionally, recent studies have shown that PGRN co-localizes with Cat D in neurons [24], and that PGRN and Cat D co-precipitate in vitro [24, 25]. We now report, for the first time, that Cat L is a lysosomal protease that localizes with and proteolytically processes and degrades intracellular PGRN in lysosomes.

## Materials and methods

### Cell culture

Human embryonic kidney cells 293 (HEK293) were cultured in growth medium (DMEM supplemented with 10% fetal bovine serum (FBS) and 1% penicillin streptomycin (PS) mix) in humidified incubators with 5% CO_2_. To knockdown PGRN, HEK293 cells were transfected with siRNA targeting the coding region of the *GRN* mRNA (Qiagen FlexiTube SI03108581) or a non-targeting siRNA control (Qiagen Negative Control siRNA 1,027,310). To establish Cat B, Cat L and Cat D stable cell lines, validated human cDNA clones (OriGene) were used to amplify the corresponding cDNA. The amplified cDNA was then subcloned into the pCDNA4-V5His6 vector (ThermoFisher Scientific) using the NheI and XhoI restriction sites. After transfection, positive clones were selected by zeocin (250 μg/ml) for 2 weeks. At least 50 colonies were pooled together to generate polyclonal stable lines for subsequence analysis. N-terminal or C-terminal 6-His tagged PGRN expression constructs (1–593) were previously described [11]. Recombinant PGRN protein (N-terminal 6 histidines tag) used for cell treatment was produced by affinity purification [11].

Neurons differentiated from induced pluripotent stem cells (iPSCs) were generated and maintained as previously described [26].

### In vitro proteolytic reactions

**Fig. 1:**
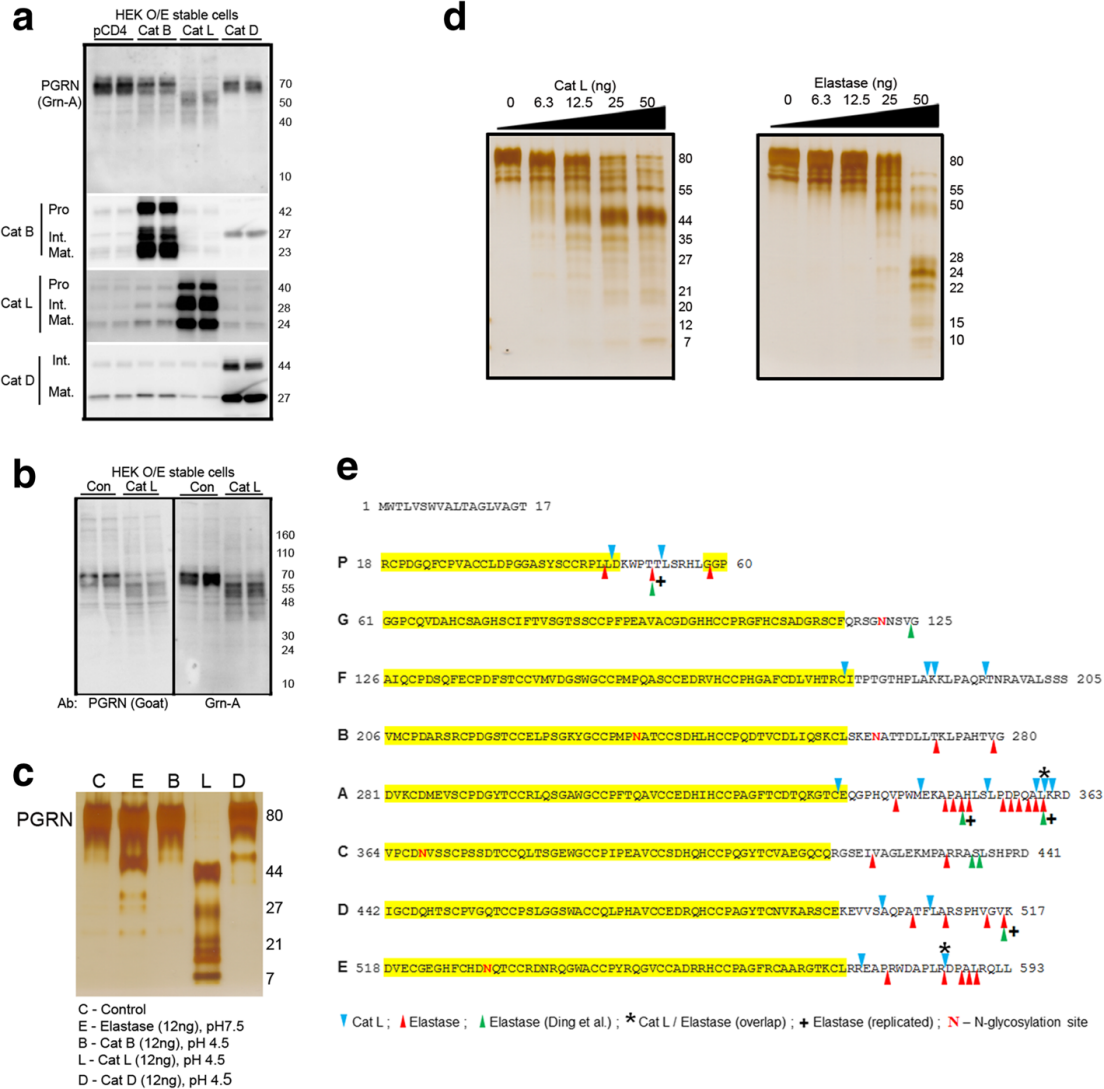
Cathepsin L proteolytically processes intracellular PGRN, and in a manner distinct from elastase. **a** HEK293 stable cell lines stably overexpressing pCDNA4 (pCD4), Cat B, Cat L and Cat D were analyzed for levels of PGRN and its fragments by western blot using Grn-A antibody. The expression levels of different forms (pro-, intermediate- and mature- [40, 41]) of Cat B, Cat L and Cat D were validated in the stable cell lines. **b** A second, full-length PGRN polyclonal goat antibody was applied to detect PGRN and its proteolytic fragments in HEK-Cat L cells in comparison to HEK-pCDNA4 cells (Con). **c** Recombinant PGRN was incubated with same amount of elastase (E), Cat B (B), Cat L (L) or Cat D (D) optimal reaction conditions. Cat L, but not Cat B or Cat D, processed PGRN into protein fragments after the reaction. **d** Recombinant PGRN protein products were detected after the addition of increasing concentrations of recombinant Cat L (*left panel*) or elastase (*right panel*). **e** Cleavage sites used by Cat L and elastase on PGRN were mapped by LC/MS analysis of the small peptides produced from the in vitro proteolytic reaction. All detected peptides are listed in Tables 1 and 2. Annotated MS/MS spectra of the identified granulin peptides were included in Additional file 1: Figure S2. Cat L and elastase cleavage sites are labeled by *blue triangle* and *red triangle*, respectively. Common cleavage sites are highlighted by the *asterisk* sign. Previously published elastase cleavage sites are labeled by *green triangle*. Elastase cleavage sites replicated from the current study are highlighted by plus sign. N-glycosylation sites were highlighted in *red*

One microgram of PGRN protein was mixed with vehicle or the indicated amount of recombinant human Cat L, Cat B or Cat D (R&D systems), or purified neutrophil elastase (Athens Research & Technology), followed by incubation at 37 °C for 15 h (Fig. 1b, c) or for 1 h (Additional file 1: Figure S2). Cat L reactions were carried out in a buffer containing 50 mM MES, 5 mM DTT, 1 mM EDTA adjusted to pH 4.5. Elastase reactions were carried out in a buffer containing 100 mM Tris–HCl, 500 mM NaCl, pH 7.5. For Cat D reaction, 20 μg/ml of Cat D was pre-activated in a buffer containing 100 mM NaOAc, 150 mM NaCl, pH 4.5 buffer at 37 °C for 30 min. After pre-activation, the specified Cat D amount was applied for reaction in the same buffer. For Cat B reaction, 10 μg/ml of Cat B was pre-activated in a buffer containing 25 mM MES, 5 mM DTT, pH 5 buffer at 25 °C for 15 min. After pre-activation, the specified Cat B amount was applied for reaction in a buffer with 25 mM MES, pH 4.5. Cat L inhibitor I, Z-Phe-Phe-Fluoromethylketone (Z-FF-FMK), was purchased from Santa Cruz Biotechnology.

### Analysis of cysteine cathepsin activity by BMV109 probe

HEK293 cells were treated with BMV109 [27] (1/500×) diluted in growth medium for 1 h. Labeled cells were washed with PBS, fixed in 4% paraformaldehyde at 37 °C for 15 minutes, followed by permeabilization with 1% triton-X in PBS for 10 minutes. To demonstrate colocalization of active cysteine cathepsin activity with other proteins, BMV109 probe labeled cells were co-labeled with other antibodies as describled in the immunocytofluorescence analysis section below. 

### Liquid chromatography-mass spectrometry (LC-MS)

PGRN was proteolytically processed by recombinant Cat L (R&D Systems) or elastase (Athen’s Research) for 16 h by following manufacturer’s instructions. PGRN protein in Cat L reaction buffer without protease was included as a negative control. The reaction products were lyophilized, resuspended and desalted using a 10 μl C18 tip (Pierce/ThermoFisher Scientific). Purified peptides were then dissolved in 20 μl of a solution composed of 2.5% acetonitrile (CH_3_CN) and 2.5% formic acid (FA) in water for subsequent liquid chromatography-mass spectrometry (LC-MS) analysis. LC-MS based peptide identification was performed on the Q-Exactive mass spectrometer coupled to an EASY-nLC (Thermo Fisher Scientific). Five microliters of the peptides were loaded onto a 100 μm × 120 mm capillary column packed with Halo C18 (2.7 μm particle size, 90 nm pore size, Michrom Bioresources) at a flow rate of 300 nl min^−1^. Peptides were separated using a gradient of 2.5–35% CH_3_CN/0.1% FA for 45 min, 35–100% CH_3_CN/0.1% FA for 1 min and then 100% CH_3_CN/0.1% FA for 8 min, followed by an immediate return to 2.5% CH_3_CN/0.1% FA and a hold at 2.5% CH_3_CN/0.1% FA. Peptides were introduced into the mass spectrometer via a nanospray ionization source and a laser pulled ~3 μm orifice with a spray voltage of 2.0 kV. Mass spectrometry data was acquired in a data-dependent “Top 10” acquisition mode with lock mass function activated (*m/z* 371.1012; use lock masses: best; lock mass injection: full MS), in which a survey scan from *m/z* 350–1600 at 70, 000 resolution (AGC target 1e^6^; max IT 100 ms; profile mode) was followed by 10 higher-energy collisional dissociation (HCD) tandem mass spectrometry MS/MS scans of the most abundant ions at 17,500 resolution (AGC target 5e^4^; max IT 100 ms; centroid mode). MS/MS scans were acquired with an isolation width of 1.6 *m/z* and a normalized collisional energy of 26%. Dynamic exclusion was enabled (peptide match: preferred; exclude isotopes: on; underfill ratio: 1%; exclusion duration: 30 s). Product ion spectra were searched using the SEQUEST and Mascot search engines on Proteome Discoverer 1.4 (Thermo Fisher Scientific) against the human granulin sequence. Search Parameters were as follows: no enzyme (unspecific), maximum missed cleavages = 2, and peptides MW between 350 and 5000; mass tolerance at 20 ppm for precursor ions and at 0.02 Da for fragment ions, and dynamic modifications on methionines (+15.9949 Da: oxidation). The result files were then further analyzed by Scaffold 4.3 (Proteome Software) and the annotated MS/MS spectra of the peptides identified by both SEQUEST and Mascot were filtered by minimal XCorr cut-off values (1.3 for singly and doubly charged peptides; 2.5 for triply charged peptides) and mascot Ion Score value of 20 and then manually evaluated. The XCorr scores cut-offs were set at relatively low values since most of the cleaved peptides observed are short in length. Annotated MS/MS spectra of the identified granulin peptides were included in Additional file 1: Figure S2. Peptides identified from the non-digested negative control were considered as background and were therefore subtracted from the peptide list generated by Cat L and elastase.

### Protein extraction, SDS-PAGE, and Western blotting (WB)

Cells were lysed by adding M-Per mammalian protein extraction reagent (ThermoFisher Scientific) supplemented with a protease inhibitor cocktail (1% *v*/v) (EMD Millipore) directly to culture plates placed on ice for 15 min with occasional mixing. Insoluble cell debris and proteins were removed by centrifugation at 16,000 × g at 4 °C for 10 min. The M-Per soluble fraction was used for subsequent Western blotting analysis.

Protein concentrations of cell lysates were determined by BCA assay. Normalized protein was mixed with equal volume of laemmli sample buffer (2×) containing 5% (*v*/v) β-mercaptoethanol followed by heating for 2 min at 85 °C. Samples were resolved using Tris-glycine 4–20% gradient gels (ThermoFisher Scientific). For WB, proteins were transferred onto PVDF membranes followed by standard blocking and antibody probing procedures. Total protein staining of gels was performed using a mass spectrometry compatible silver staining kit (ThermoFisher Scientific).

### Immunocytofluorescence (ICF) analysis

Cells were grown on poly-L-lysine-coated glass coverslips. Cells were fixed in 4% paraformaldehyde in PBS at 37 °C for 15 min followed by permeabilization with 1% triton-X 100 in PBS for 10 min. Cells were then blocked in 5% non-fat milk in PBS for one hour at room temperature, followed by incubation in primary antibodies (in blocking solution) for 1 h at room temperature. After washing cells in PBS twice for 10 min each, secondary antibodies conjugated to Alexa Fluor® dye (in blocking solution) were added and incubated for 1 h protected from light. After washing with PBS, cell nuclei were stained with Hoechst 33,342 (1/10,000× in PBS) for 15 min prior to mounting cells onto glass slides with ProLong® Gold antifade mountant (Invitrogen) and dried for at least 16 h at room temperature in the dark. Images were captured using a Zeiss LSM 880 laser scanning confocal microscope.

### Immunohistofluorescence analysis (IHF)

Frontal cortical tissue sections were rehydrated in a graded series of xylene and alcohol prior to antigen retrieval for 30 min in 10 mM sodium citrate, pH 6 buffer with 0.05% tween 20. Sections were blocked in Dako serum-free protein Block reagent (Agilent Technologies, X0909) for 1 h at room temperature, followed by a 1 h incubation with primary antibodies diluted in Dako Antibody Diluent (Agilent Technologies, S2022) at room temperature. After 3 washes with PBS, sections were incubated with secondary antibodies conjugated with Alexa Fluor® dye diluted in Dako Antibody Dilutent for 1 h followed by 3 PBS washes. Sections were mounted using Vectashield mounting medium with DAPi and coverslipped. Mounted sections were dried for at least 16 h at room temperature in the dark prior to image analysis using a Zeiss LSM 880 laser scanning confocal microscope.

### Antibodies

The Grn-A antibody (Novus, 26,320,002) was used for ICF (1:100), IHF (1:100), IHC (1:500) and WB (1:1000). The polyclonal goat PGRN antibody (R&D systems, AF2420) was used for WB (1:1000). Cat L antibody (R&D Systems, AF952) was used for ICF (1:200), IHF (1:500) and WB (1:2000). Lamp2 antibody (BD Biosciences, clone H4B4) was used for ICF (1:300). Lamp1 antibody (Santa Cruz, clone E-5) was used for IHF (1:100). NeuN (EMD Millipore, clone A60) was used for IHF (1:100). Cathepsin D polyclonal antibody (Athens Research & Technology, 01-12-030104) was used for WB (1:2000). The cathepsin D antibody (Santa cruz, clone C-20) was also used for WB (1:200). The cathepsin B antibody (R&D systems, AF953) was used for WB (1:1000). GAPDH antibody (Meridian, H86504M) was used for WB (1: 10,000).

### Lysosome purification

Lysosome purification was performed by magnetic fractionation with minor modifications [28]. In brief, HEK293 cells grown in 6-well culture plates were treated with *GRN* transcript-specific siRNA for 72 h. The culture medium was then replaced with medium containing dextran-coated magnetic nanoparticles (FluidMAG-DX; Chemicell) at a concentration of 0.5 mg/ml. After 6 h, cells were washed three times with PBS, fresh growth medium was added, and cells were cultured for an additional hour under normal cell culture condition. Cells were then washed twice in cold PBS and lysed in 400 μl hypotonic buffer (10 mM HEPES pH 7.4, 10 mM NaCl, 5 mM MgCl2 and protease inhibitor cocktails (1% *w*/*v*)) for 20 min on ice. The lysed cells were then sheared with a dounce homogenizer for 10 s in a microfuge tube, which was then placed in a microfuge tube magnetic separator. After 10 min, cell nuclei were removed and the magnetic fraction containing lysosomes was retained. The lysosomal fraction was washed twice with hypotonic buffer. The washed magnetic fraction was then lysed in M-Per protein extraction buffer (ThermoFisher Scientific) and normalized to protein amount by BCA assay before being analyzed by WB.

### Approvals

For studies using human postmortem tissues, autopsy consent was given by the next-of-kin and studies were conducted with approval of the Institutional Review Board at Mayo Clinic Florida.

## Results and discussion

### Cathepsin L is a specific lysosomal PGRN protease that cleaves PGRN into granulins

We first confirmed that PGRN is localized to the lysosome (Additional file 1: Figure S1a, b) and that the PGRN antibody was specific as shown by siRNA-mediated PGRN knockdown (Additional file 1: Figure S1a). To determine whether cathepsin cysteine protease(s) might be responsible for intracellular PGRN proteolytic processing within the lysosome, we generated stable HEK293 lines overexpressing (OE) cathepsin B (Cat B), Cat L or Cat D. While Cat B-OE or Cat D-OE cells showed only minor, non-specific, reductions in PGRN levels, Cat L-OE cells showed a shift from the predominantly 70 kDa PGRN to lower-molecular weight species (40 to 55 kDa) (Fig. 1a). This shift to lower molecular weight PGRN species upon exposure to Cat L-OE was confirmed using a second full-length PGRN antibody (Fig. 1b). These results strongly suggest that Cat L is a specific intracellular PGRN protease involved in regulating the maturation and turnover of PGRN within the lysosome.

To further validate the specificity of Cat L on PGRN, we performed in vitro protease reactions on recombinant PGRN with the same amount of recombinant Cat B, Cat L or Cat D at lysosomal acidic pH under a saturated timepoint of 15 h. Recombinant neutrophil elastase (NE) was included as a positive control. Consistent with our observation in a human cell system, only Cat L, but not Cat B or Cat D, cleaved PGRN into protein products similar in size to poly-granulin (~44 kDa to ~20 kDa) and mono-granulin (~10 kDa) fragments. PGRN was also processed by NE to form a 40–50 KDa fragment under the current reaction conditions (Fig. 1c). At a shorter time point of 1 h, Cat L also efficiently processed PGRN into poly-granulin sized fragments at pH 4.5. Under the same reaction condition, co-incubation with different concentrations of Z-FF-FMK, a Cat L inhibitor, blocked PGRN processing by Cat L in a dose-dependent manner (Additional file 1: Figure S2). These results further support Cat L, rather than Cat B or Cat D, as a potential PGRN protease in the lysosome.

Previous work demonstrated that NE, a predominantly extracellular protease, could cleave PGRN into poly-granulins and granulins, cleaving PGRN exclusively at linker regions to release granulins at 7 cleavage sites on PGRN [18]. To elucidate how the lysosomal protease Cat L cleaves PGRN, and how its cleavage activity differs from that of NE, we performed an in vitro PGRN cleavage analysis using recombinant Cat L and NE under acidic (pH 4.5) and neutral (pH 7) conditions, respectively, for 15 h. Both Cat L and NE cleaved PGRN dose-dependently, but the cleavage products generated were distinct (Fig. 1d). Liquid chromatography-mass spectrometry (LC-MS)-based proteomic analyses of the Cat L and NE small peptide products confirmed that Cat L and NE specifically and distinctively cleaved PGRN at multiple linker regions as well as protein regions proximal to granulin/linker boundaries, with only two of the 16 Cat L cleavage sites shared with NE (Fig. 1e). Cat L sites were evenly distributed on linker regions between granulin P-G, F-B, A-C, D-E and on C-terminal end, while no Cat L sites were found on the linker G-F and B-A (Tables 1 and 2 and Additional file 1: Figure S3) which is likely due to interference by N-glycosylation modifications [29]. Of note, we also detected 4 out of the 7 NE cleavage sites identified by Ding and colleagues [18], further validating the efficacy of our approach to map protease cleavage sites.

**Table 1:** Summary of identified peptides from Cat L proteolytic processing of PGRN

	Peptide	Sequence location	QS	SEQUEST XCorr	Mascot Ion Score	Δ mass (ppm)
(i) DKWPTT (Linker P – G)						
CL-1	DKWPTT	47–52	2	1.46	20	−0.39
(ii) ITPTGTHPLAKKLPAQR (Linker F – B)						
CL-2	ITPTGTHPLA	179–188	2	1.33	23	0.65
CL-3	KKLPAQR	189–195	2	1.78	31	−0.43
CL-4	KLPAQR	190–195	2	1.49	31	−0.29
(iii) EQGPHQVPWM (Linker A – C)						
CL-5	EQGPHQVPWM(ox)	336–345	2	1.54	23	0.23
(iv) LPDPQALK (Linker A – C)						
CL-6	LPDPQALK	354–361	2	1.73	36	0.20
CL-7	LPDPQAL	354–360	2	1.86	30	−0.64
CL-8	LPDPQA	354–359	1	1.57	30	−0.26
(v) AQPATF (Linker D – E)						
CL-9	AQPATF	502–507	1	1.47	22	0.53
(vi) EAPRWDAPLR (Linker C-terminus end)						
CL-10	EAPRWDAPLR	576–585	3	1.83	25	0.13

QS: Charge state, (ox) methionine oxidation, Δ mass (ppm), difference between experimental and theoretical masses in parts-per-million. Annotated MS/MS spectra of the identified granulin peptides generated by Cat L activity were included in Additional file 1: Figure S2, numbered from CL-1 to CL-10

**Table 2:** Summary of identified peptides from elastase proteolytic processing of PGRN

	Peptide	Sequence location	QS	SEQUEST XCorr	Mascot Ion Score	Δ mass (ppm)
(i) LDKWPTLSRHLG (Linker P – G)						
EL-1	LDKWPT	45–50	2	1.33	29	−0.58
EL-2	TLSRHLG	51–57	2	1.43	24	−0.62
(ii) KLPAHTV (Linker B – A)						
EL-3	KLPAHTV	273–279	2	1.42	29	−0.33
(iii) PWMEKAPAHLSLPDPQAL (Linker A – C)						
EL-4	PWMEKAPA	343–350	2	1.73	23	0.55
EL-5	PWM(ox)EKA	343–348	2	1.37	28	−1.70
EL-6	AHLSLPDPQ	350–358	2	1.93	23	−0.57
EL-7	HLSLPDPQAL	351–360	2	3.22	70	1.3
EL-8	HLSLPDPQA	351–359	2	1.81	36	1.2
EL-9	HLSLPDPQ	351–358	2	1.97	38	0.79
EL-10	HLSLPDP	351–357	2	1.61	40	−1.3
EL-11	HLSLP	351–355	1	1.34	23	1.0
EL-12	LSLPDPQ	352–358	1	1.75	35	0.23
(iv) VAGLEKMPA (Linker (Linker C – D)						
EL-13	VAGLEKM(ox)PA	423–431	2	1.41	32	0.79
(v) TFLARSPHVGV (Linker D – E)						
EL-14	TFLARSPHV	506–514	2	1.89	34	0.53
EL-15	RSPHVGV	510–516	2	1.82	33	−0.24
(vi) RWDAPLRDPAL (Linker C-terminus end)						
EL-16	RWDAPLRDPAL	579–589	2	2.55	21	−0.19
EL-17	RWDAPLRDPA	579–588	3	2.94	50	2.0
EL-18	RWDAPLRDP	579–587	2	2.38	24	0.52
EL-19	RWDAPLR	579–585	2	1.98	26	−1.1

QS: Charge state, M(ox) methionine oxidation, Δ mass (ppm), difference between experimental and theoretical masses in parts-per-million. Annotated MS/MS spectra of the identified granulin peptides generated by elastase activity were included in Additional file 1: Figure S2, numbered from EL-1 to EL-19

Little was previously known about PGRN lysosomal biology, partially due to a lack of understanding in how PGRN traffics within endosomal/lysosomal compartments and the functional molecular form(s) of PGRN in the lysosome. Our data confirm that Cat L exclusively cleaves PGRN on the linker regions to release poly-granulin and mono-granulin fragments under the acidic conditions found within lysosomes. These data provide strong evidence to support Cat L as a novel intracellular PGRN-to-granulin convertase, analogous to the functional roles of NE [18] and MMP12 [19] as extracellular PGRN proteases.

### PGRN co-localizes with active Cat L in lysosomes

**Fig. 2:**
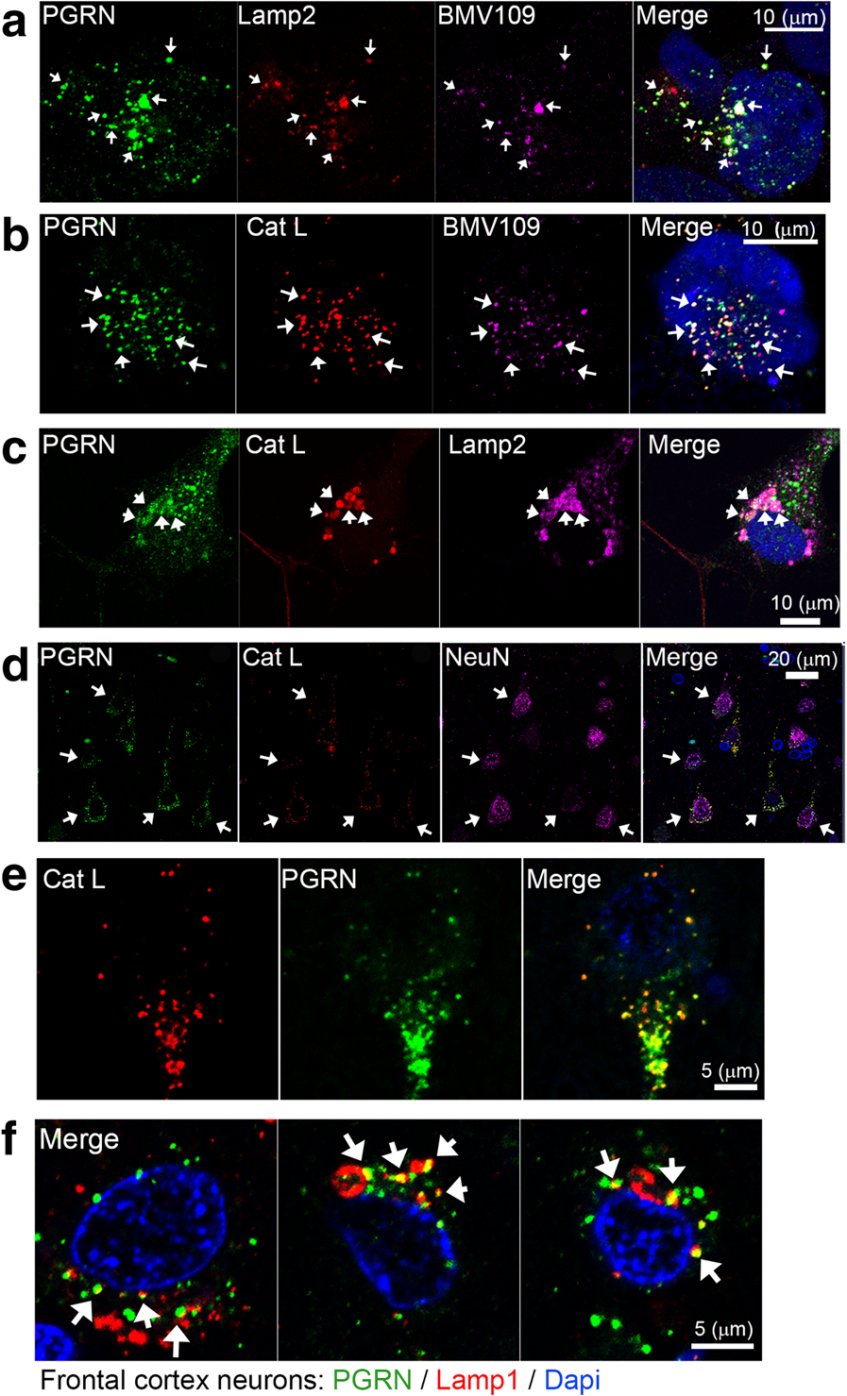
Cellular PGRN co-localize with Cat L and active cysteine cathepsins in lysosomes. **a** Immunofluorescence (IF) analysis of HEK293 cells showing the localization of PGRN (*green*), Lamp2 (*red*) and active cysteine cathepsins (BMV109 activity probe; *violet*). Nuclei in merged images were visualized by Hoechst 33,342 staining. **b** IF analysis of PGRN (*green*), Cat L (*red*) and BMV109 (*violet*) in HEK293 cells. **c** IF analysis of human wild-type iPSC-neurons showing the localization of PGRN (*green*), Cat L (*red*), and Lamp2 (*violet*). **d** IF analysis of neuronal cells in the frontal cortex of a sporadic FTLD patient showing the localization of PGRN (*green*), Cat L (*red*), and NeuN (*violet*). **e** High magnification IF analysis of Cat L (*red*) and PGRN (*green*) in a cortical neuron from the frontal cortex. **f** High magnification IF analysis of 3 representative cortical neurons in the frontal cortex of a sporadic FTLD patient showing the localization of PGRN (*green*), Lamp1 (*red*), and the nucleus (DAPI; *blue*). The *white arrows* show triple co-localization (**a** – **c**) or co-localization of PGRN and Cat L in NeuN position cells (**d**)

To confirm that Cat L acts on PGRN in human cells*,* we analyzed the subcellular localization of endogenous PGRN, Lamp2 and Cat L in HEK293 cells, induced pluripotent stem cell (iPSC)-derived neurons [26] and frontal cortex tissue from a sporadic FTLD patient (negative for mutations in *GRN*). Immunofluorescence (IF) results demonstrate that intracellular PGRN co-localizes with Lamp2 and Cat-L in HEK293 and iPSC-derived neurons (Fig. 2a–c), as well as PGRN and Cat L co-localization in NeuN-positive neurons (Fig. 2d and e) and partial co-localization of PGRN and Lamp2 (Fig. 2f) in human frontal cortex. These data provide strong indication that PGRN and Cat L co-localize within lysosomes. Using BMV109, a quenched fluorescence activity-based probe (qABP) that specifically labels active cysteine cathepsins in live cells [27], we found triple co-localization between PGRN, BMV109 and Cat L indicating that PGRN co-localized with active Cat L in lysosomes (Fig. 2a and b). Collectively, these data support the notion that PGRN is physiologically targeted to the lysosome, where Cat L acts as an intracellular PGRN protease within lysosomes.

## Conclusions

Previous studies of PGRN in dementia largely focused on the neurotrophic functions of full-length extracellular PGRN. Given the predominantly lysosomal localization of PGRN, and that it remains unclear whether full-length and/or cleaved granulins play more important biological roles, we focused here on exploring the metabolism of intracellular PGRN within the lysosome. One prior study using purified granulin and recombinant CatL suggested that, at least in vitro, CatL had the capacity to degrade PGRN [30]. Here, we provide evidence that Cat L is, in fact, a novel mediator of intracellular PGRN proteolysis, regulating levels of PGRN and its granulin fragments. Due to an entirely unique set of cleavage sites utilized by extracellular elastase and lysosomal Cat L, it is conceivable that distinct pools of poly-granulin and granulin species are present within the extracellular space versus in lysosomes. Further, extracellular PGRN and granulins possess opposing activities in inflammation and cell proliferation. Specifically, PGRN suppresses inflammation and promotes cell proliferation, whereas granulin exacerbates inflammation and inhibits cell proliferation [18, 31]. In a recent study, granulins were demonstrated to exacerbate TDP-43 toxicity analyzed by impaired motor function in a *Caenorhabditis elegans* model [32]. While information about the regulation or existence of functional and physiological granulin species within the lysosome is currently lacking, our identification of Cat L-mediated PGRN cleavage fragments within the lysosome open up new opportunities to assess the function(s) of endogenous lysosomal granulin species.

Though little is known about neuronal functions of Cat L, Cat L knockout mice implicate Cat L in the production of brain neurotransmitters including neuropeptide Y [33] and dynorphin [34]. Cat L also proteolytically cleaves dynamin-1 (Dnm1), a protein that plays a key role in the fission reaction of synaptic vesicle endocytosis and synaptic transmission at nerve terminals [35] and is involved in the pathogenesis of proteinuric kidney disease [36]. Our data identify Cat L as the first lysosomal protease for PGRN, although future studies are needed to assess whether cleavage of PGRN by Cat L acts as a rate-limiting step in PGRN processing, facilitating subsequent cleavage events by other lysosomal proteases. In addition, while the data presented in the current report are suggestive of a key role for Cat L in PGRN processing in neurons, additional studies performed in Cat L knockout animals will be an intriguing new research direction to further clarify the mechanisms by which PGRN is processed within neurons in the brain. The elucidation of consequences on PGRN processing following altered expression of the endogenous cathepsin L inhibitors, cystatin C and M/E [37, 38], will also be an interesting research direction for future studies, and may represent a novel approach to therapeutically modulate the PGRN/granulin axis in vivo. In conclusion, as Cat L-mediated processing of PGRN in the lysosome may be of critical importance in the neuropathobiology of FTLD-GRN, the current findings provide a first step in understanding the physiological importance of Cat L processing of PGRN in neurons.

## Additional file

Additional file 1:
**Figure S1.** (a) Isolated lysosomes and total lysate from HEK293 cells treated with non-targeting (Con) or *GRN* transcript targeting siRNA were analyzed for PGRN-specific signal by western blot using the goat polyclonal PGRN antibody. (b) The same samples were also analyzed for GAPDH and lysosomal markers, Lamp2 and Cat D. **Figure S2.** Cat L (5 ng) efficiently processed PGRN (50 ng) into poly-granulin fragments in 1 h reaction time under pH 4.5. Under the same reaction condition, co-incubation with Cat L inhibitor, Z-FF-FMK blocked proteolytic processing of PGRN by Cat L in a dose-dependent manner. **Figure S3.** Annotated MS/MS spectra of the identified granulin peptides shown in Fig. 1d and in Tables 1 and 2. The Proteome Discoverer result files (.msf) were imported into the Scaffold 4.3 (Proteome Software) for sequence annotation. The peptides that were identified by both SEQUEST and Mascot above the cutoff values (SEQUEST: 1.3 for singly and doubly charged peptides, 2.5 for triply charged peptides; and Mascot Ion Score: 20) were manually evaluated PGRN peptides generated by Cat L activity were designated as CL-1 to CL-10; whereas the PGRN peptides generated by elastase activity were designated as EL-1 to EL-19. The fact that all the measured precursor masses of the identified peptides were within or around 1 ppm of the theoretical masses and that the tandem mass spectra (MS/MS) exhibit a continuous stretch of b- or y- ion series, or clear peak assignments, indicating confident identifications. Sequence assignments were further supported by multiple spectra of successive cleavages of the same sequence. (DOCX 6702 kb)
