# Supplementary material for: The lysosomal protein cathepsin L is a progranulin protease
Source: Mol Neurodegener. 2017 Jul 25;12:55. doi: 10.1186/s13024-017-0196-6 (PMC5526245; doi:10.1186/s13024-017-0196-6)

The Lysosomal Protein Cathepsin L is a Progranulin Protease

**Lee et al.**

**Additional file 1**

**Figure S1** (a) Isolated lysosomes and total lysate from HEK293 cells treated with non-targeting (Con) or *GRN* transcript targeting siRNA were analyzed for PGRN-specific signal by western blot using the goat polyclonal PGRN antibody. (b) The same samples were also analyzed for GAPDH and lysosomal markers, Lamp2 and Cat D.

**Figure S2**. Cat L (5ng) efficiently processed PGRN (50ng) into poly-granulin fragments in 1 hour reaction time under pH 4.5. Under the same reaction condition, co-incubation with Cat L inhibitor, Z-FF-FMK blocked proteolytic processing of PGRN by Cat L in a dose-dependent manner.

**Figure S3** Annotated MS/MS spectra of the identified granulin peptides shown in **Fig. 1d** and in **Tables 1** and **2**. The Proteome Discoverer result files (.msf) were imported into the Scaffold 4.3 (Proteome Software) for sequence annotation. The peptides that were identified by both SEQUEST and Mascot above the cutoff values (SEQUEST: 1.3 for singly and doubly charged peptides, 2.5 for triply charged peptides; and Mascot Ion Score: 20) were manually evaluated PGRN peptides generated by Cat L activity were designated as CL-1 to CL-10; whereas the PGRN peptides generated by elastase activity were designated as EL-1 to EL-19. The fact that all the measured precursor masses of the identified peptides were within or around 1 ppm of the theoretical masses and that the tandem mass spectra (MS/MS) exhibit a continuous stretch of b- or y- ion series, or clear peak assignments, indicating confident identifications. Sequence assignments were further supported by multiple spectra of successive cleavages of the same sequence.

**Figure S1**


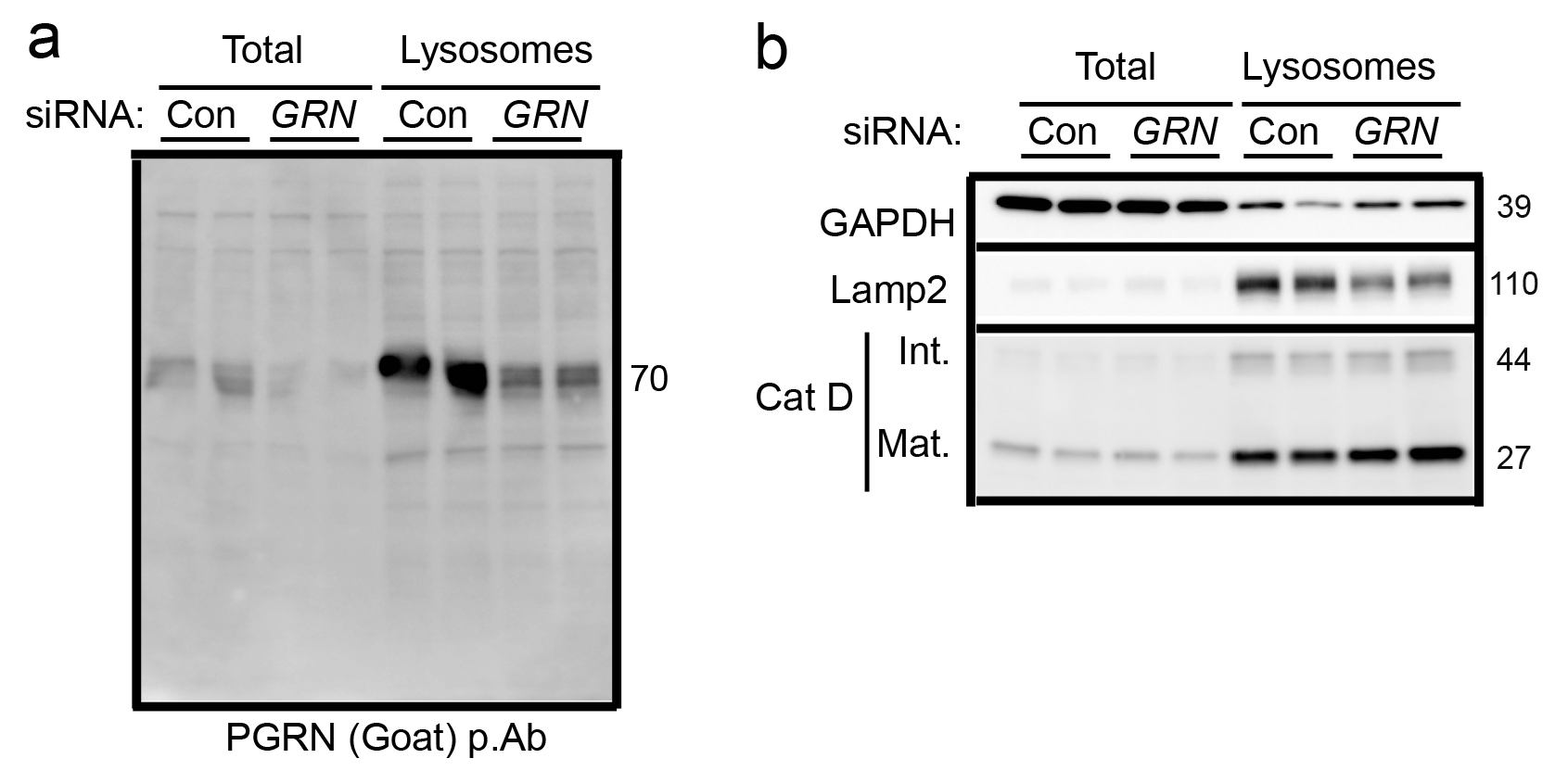


**Figure S2**

**
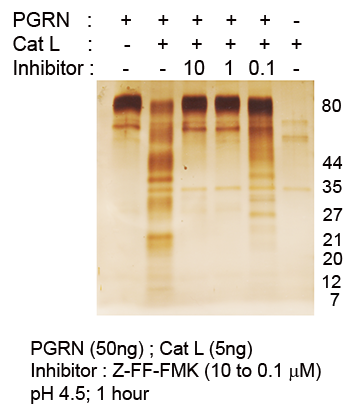
**

**Figure S3**


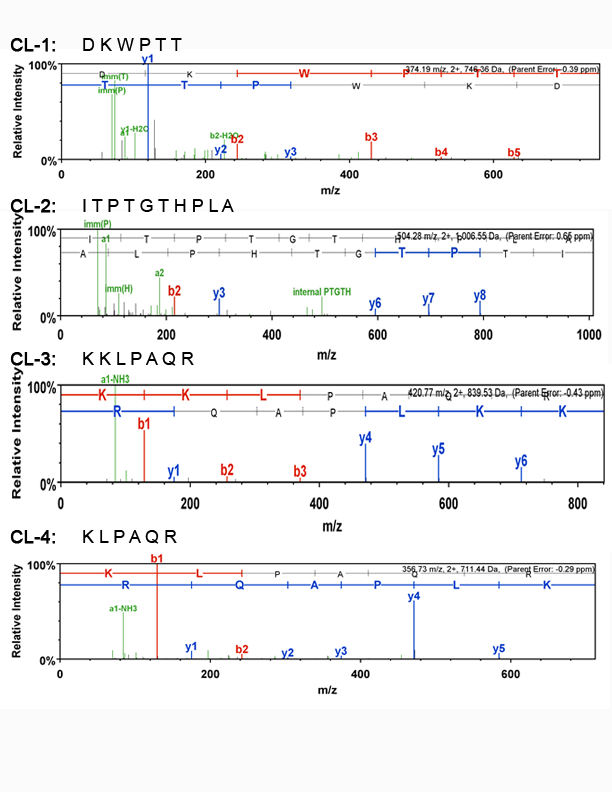


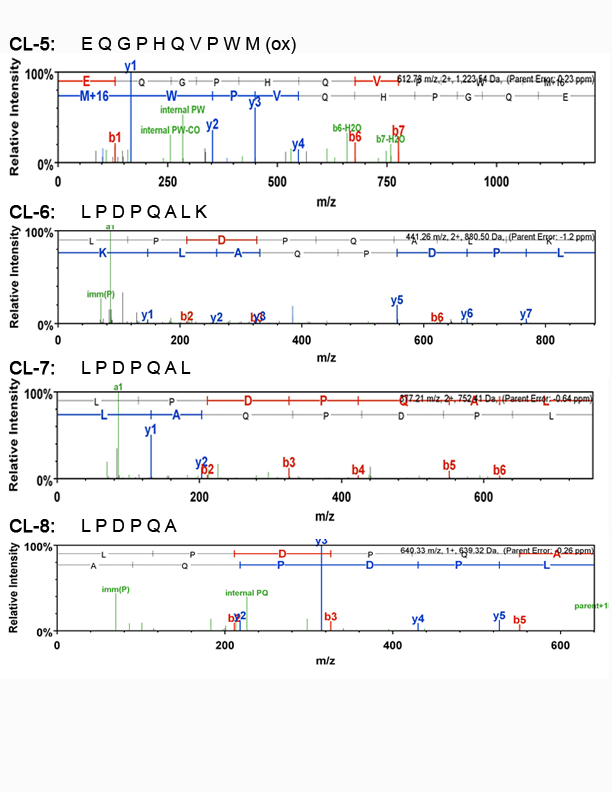


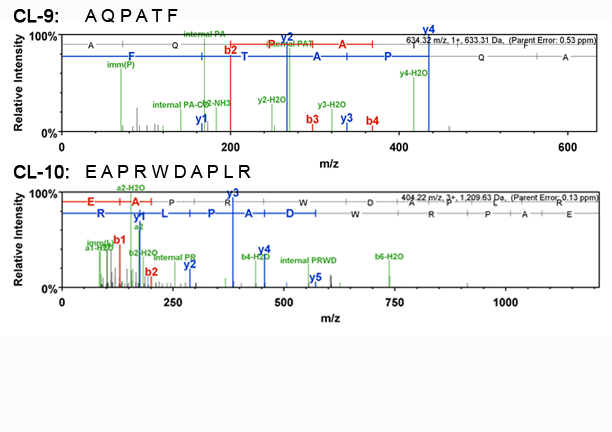


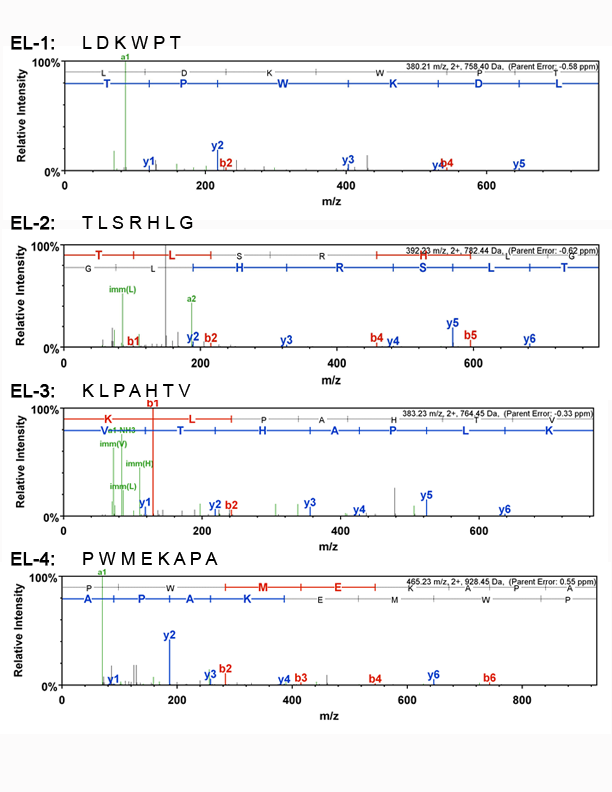


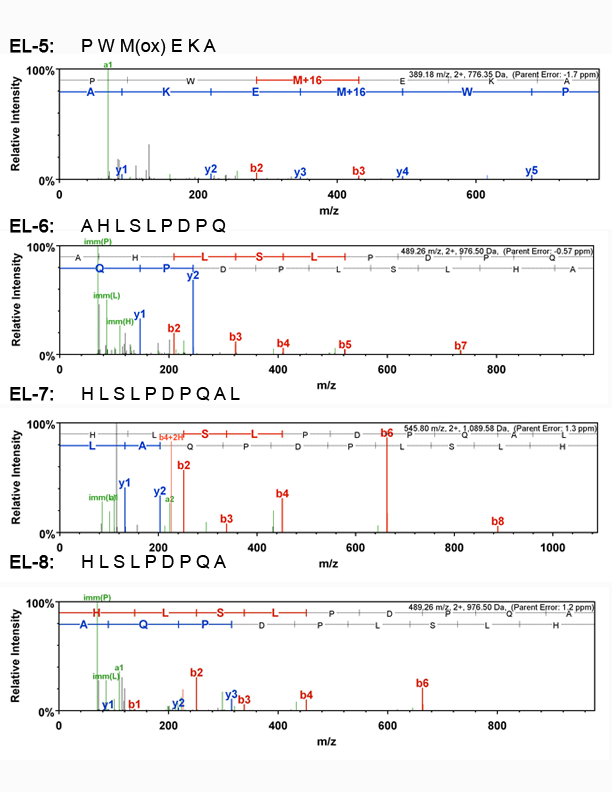


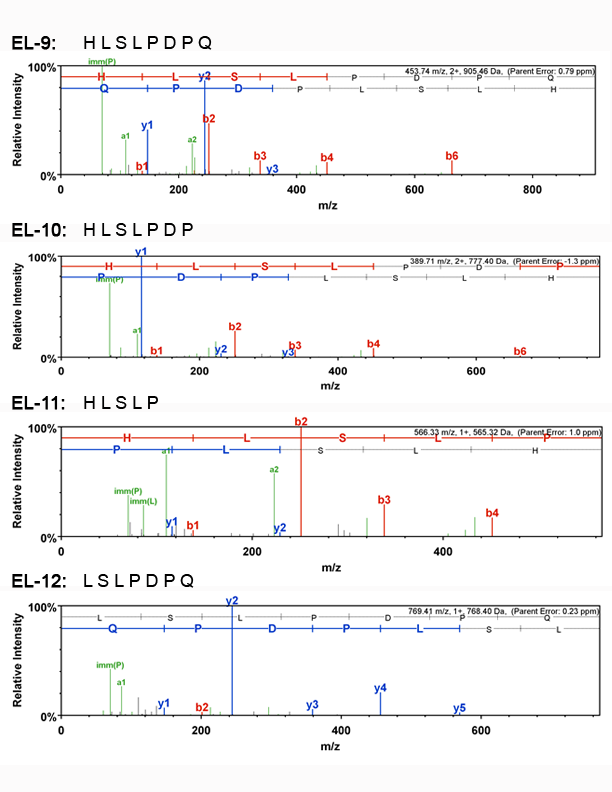


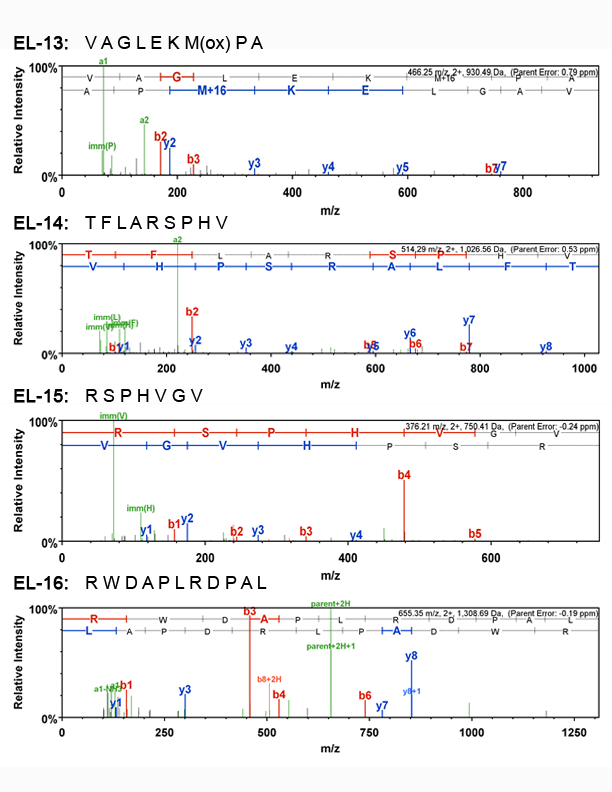

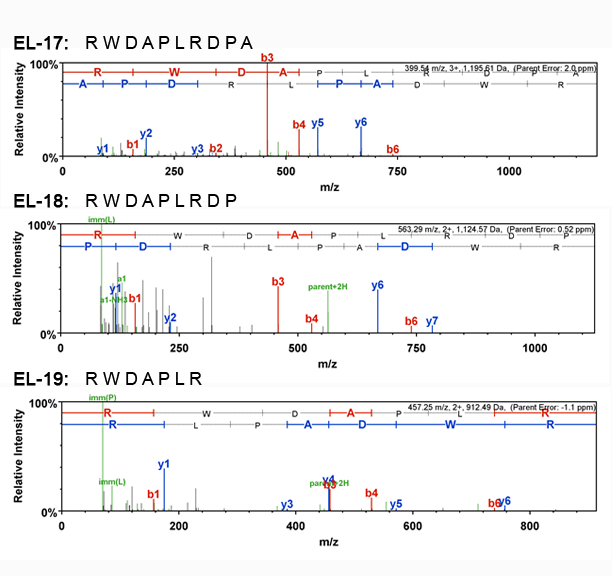

Supplement: Additional file 1: — Figure S1. (a) Isolated lysosomes and total lysate from HEK293 cells treated with non-targeting (Con) or GRN transcript targeting siRNA were analyzed for PGRN-specific signal by western blot using the goat polyclonal PGRN antibody. (b) The same samples were also analyzed for GAPDH and lysosomal markers, Lamp2 and Cat D. Figure S2. Cat L (5 ng) efficiently processed PGRN (50 ng) into poly-granulin fragments in 1 h reaction time under pH 4.5. Under the same reaction condition, co-incubation with Cat L inhibitor, Z-FF-FMK blocked proteolytic processing of PGRN by Cat L in a dose-dependent manner. Figure S3. Annotated MS/MS spectra of the identified granulin peptides shown in Fig. 1d and in Tables 1 and 2. The Proteome Discoverer result files (.msf) were imported into the Scaffold 4.3 (Proteome Software) for sequence annotation. The peptides that were identified by both SEQUEST and Mascot above the cutoff values (SEQUEST: 1.3 for singly and doubly charged peptides, 2.5 for triply charged peptides; and Mascot Ion Score: 20) were manually evaluated PGRN peptides generated by Cat L activity were designated as CL-1 to CL-10; whereas the PGRN peptides generated by elastase activity were designated as EL-1 to EL-19. The fact that all the measured precursor masses of the identified peptides were within or around 1 ppm of the theoretical masses and that the tandem mass spectra (MS/MS) exhibit a continuous stretch of b- or y- ion series, or clear peak assignments, indicating confident identifications. Sequence assignments were further supported by multiple spectra of successive cleavages of the same sequence. (DOCX 6702 kb) [file 13024_2017_196_MOESM1_ESM.docx]
